# Supplementary material for: Content-rich biological network constructed by mining PubMed abstracts
Source: BMC Bioinformatics. 2004 Oct 8;5:147. doi: 10.1186/1471-2105-5-147 (PMC528731; doi:10.1186/1471-2105-5-147)
Supplement: Additional File 5 — The original Chilibot query results of the term "long-term potentiation (LTP)" and 22 other terms, limiting the latest references analyzed to the years 1990, 1995, 2000, and 2004. [file 1471-2105-5-147-S5.bz2 › chilibotAdditionalFile5/ltp2000/html/AMPA_CAMKII.html]

 


 **AMPA** and **CAMKII** 
  
Found 36 abstracts in PubMed,  **30 abstracts were retrieved and analyzed**.  


---

 Search Google  |
 PDF files only 
|  EDU domain only 

---

**Interactive relationship** (e.g. stimulation, inhibition, etc)

- Long term potentiation LTP or increased activity of the calcium calmodulin dependent protein kinase II  [ **CAMKII** ]   **CaMKII**  induced delivery of tagged  **AMPA**  Rs into synapses.  Ref: 10731148 Science, 2000
- We conclude that local calcium influx through  **AMPA**  gated channels followed by activation of  **CaMkII**  is an important step for spinule retraction during dark adaptation.  Ref: 8528466 Eur J Neurosci, 1995
- Newly formed synapses may be silent unless sufficient depolarization is provided by coincident activity that could activate postsynaptic  **CaMKII** , resulting in the appearance of  **AMPA**  responses.  Ref: 8875937 Science, 1996
- Here we show that calcium independent  **CaMKII**  specifically binds to isolated postsynaptic densities PSDs, leading to enhanced phosphorylation of many PSD proteins including the alpha amino 3 hydroxy 5 methyl 4 isoxazole propionic acid  **AMPA**  type glutamate receptor.  Ref: 9153188 J Biol Chem, 1997
- This effect was not diminished by mutating the  **CaMKII**  phosphorylation site on the GluR1  **AMPA**  R subunit, but was blocked by mutating a predicted PDZ domain interaction site.  Ref: 10731148 Science, 2000
- This interaction places  **CaMKII**  not only proximal to a major source of calcium influx but also close to alpha amino 3 hydroxy 5 methylisoxazole 4 propionic acid  **AMPA**  type glutamate receptors, which become phosphorylated upon stimulation of NMDA receptors in these forebrain slices.  Ref: 10077668 Proc Natl Acad Sci U S A, 1999
- These results show that LTP and  **CaMKII**  activity drive  **AMPA**  Rs to synapses by a mechanism that requires the association between GluR1 and a PDZ domain protein.  Ref: 10731148 Science, 2000
- Arachidonic acid potentiates currents through calcium permeable  **AMPA**  receptors by interacting with a  **CaMKII**  pathway.  Ref: 10101246 Brain Res Mol Brain Res, 1999
- It is proposed that calcium entry through  **AMPA**  receptors modulates the glycine receptor function via coactivation of  **CaMKII**  and calcineurin in SDCN neurones.  Ref: 9882741 J Physiol, 1999
- Driving  **AMPA**  receptors into synapses by LTP and  **CaMKII**  requirement for GluR1 and PDZ domain interaction.  Ref: 10731148 Science, 2000
- Recent studies have shown that phosphorylation of  **AMPA**  receptors by cAMP dependent protein kinase PKA and calcium and calmodulin dependent protein kinase II  **CaMKII**  potentiates their activity, but phosphorylation of the receptor subunits may also affect their interaction with intracellular proteins, and their expression at the plasma membrane.  Ref: 11059799 Neurochem Res, 2000
- These results support biochemical observations indicating that PKC, similar to calcium calmodulin dependent protein kinase II  [ **CAMKII** ] , has an important role in controlling synaptic strength via modulation of  **AMPA**  receptor function, potentially through the direct phosphorylation of the GluR1 subunit.  Ref: 9819284 J Neurophysiol, 1998
- **CaMKII** , and calcineurin, resulting in phosphorylation of  **AMPA**  receptors by  **CaMKII**  and inactivation of NMDA receptors at least in part through calcineurin.  Ref: 10218114 Adv Second Messenger Phosphoprotein Res, 1999

**Parallel relationship** (e.g. studied together, co-existance, homology, etc.)

- Ten minutes prior to the retention test, through indwelling cannulae placed in the CA1 region of the dorsal hippocampus, they received 0.5 microl infusions of saline, a vehicle 2% dimethylsulfoxide in saline, the glutamate NMDA receptor blocker, aminophosphonopentanoic acid AP5 5.0 microg, the  **AMPA**  kainate receptor blocker, cyanonitroquinoxaline dione CNQX 0.25 or 1.25 microg, the metabotropic receptor antagonist, methylcarboxyphenylglycine MCPG 0.5 or 2.5 microg, the inhibitor of calcium calmodulin dependent protein kinase II  [ **CAMKII** ]  KN62 3.5 microg, the inhibitor of cAMP dependent protein kinase PKA, Rp cAMPs 0.1 or 0.5 microg, the stimulant of the same enzyme, Sp cAMPs 0.1 or 0.5 microg, or the inhibitor of the mitogen activated protein kinase MAPK kinase, PD098059 10 or 50 microM.  Ref: 10840135 Behav Brain Res, 2000
- These findings suggest that memory formation of spatial habituation depends on the functional integrity of NMDA and  **AMPA**  kainate receptors and  **CaMKII**  activity in the CA1 region of the hippocampus.  Ref: 11040265 Learn MemLearn Mem, 2000
- To study potential molecular mechanisms of epileptogenesis in the neocortex, the motor cortex of rats was injected with tetanus toxin TT, and gene expression for 67 kDa glutamic acid decarboxylase GAD 67, type II calcium calmodulin dependent protein kinase  **CaMKII** , NMDA receptor subunit 1 NR1, and  **AMPA**  receptor subunit 2 GluR2 was investigated by in situ hybridization histochemistry.  Ref: 9045741 J Neurosci, 1997
- Identification of the postsynaptic adapter for  **CaMKII**  fills a critical gap in the understanding of LTP because  **CaMKII**  mediated phosphorylation of  **AMPA**  receptors is an important step during LTP.  Ref: 10077668 Proc Natl Acad Sci U S A, 1999
- The recent findings of the stimulus induced translocation of calcium calmodulin dependent protein kinase II  **CaMKII**  and the alpha amino 3 hydroxy 5 methyl 4 isoxazolepropionic acid  **AMPA**  receptor to and from the postsynaptic region are model cases for understanding how the dynamic localization of signalling proteins is used to regulate signal transduction.  Ref: 10802539 Trends Cell Biol, 2000
- This suggested a close local link between  **AMPA**  kainate receptors and  **CaMKII**  indicating a possible postsynaptic function of spinules.  Ref: 9068842 Vision Res, 1996
- The phosphorylation of the glutamate receptor 1 GluR1 subunit of  **AMPA**  receptors by protein kinase A PKA, protein kinase C PKC, and calcium calmodulin dependent protein kinase II  **CaMKII**  has been characterized extensively.  Ref: 10366608 J Neurosci, 1999
- Using phosphorylation site specific antibodies, we found that induction of chem LTD produces a persistent dephosphorylation of the GluR1 subunit of  **AMPA**  receptors at serine 845, a cAMP dependent protein kinase PKA substrate... a substrate of protein kinase C PKC and calcium calmodulin dependent protein kinase II  [ **CAMKII** ]   **CaMKII** .  Ref: 9856470 Neuron, 1998
